# Lactic acid bacteria isolated from traditional Iranian butter with probiotic and cholesterol‐lowering properties: In vitro and in situ activity

**DOI:** 10.1002/fsn3.3066

**Published:** 2022-09-21

**Authors:** Mahbubeh Ostadzadeh, Mohammad B. Habibi Najafi, Mohammad R. Ehsani

**Affiliations:** ^1^ Department of Food Science and Technology Ferdowsi University of Mashhad Mashhad Iran; ^2^ Department of Food Science and Technology, Science and Research Branch Islamic Azad University Tehran Iran

**Keywords:** cholesterol, *Lactobacillus*, probiotic, traditional butter

## Abstract

Producing butter from yogurt is known as a traditional way practiced in Iran and elsewhere, particularly in rural areas. Lactic acid bacteria (LAB) with probiotic and cholesterol‐lowering properties were isolated from traditional butter collected in different regions of Iran. Then, isolates were identified and applied as adjunct culture in industrial butter production. Ten samples of traditional Iranian butter were collected from local farms. Fifty‐four isolates were considered LAB due to biochemical examinations. Molecular techniques then identified 10 strains showing high cholesterol reduction ability and tolerated bile and acid. The sequence analysis revealed that four isolates belonged to *Enterococcus durans*, four isolates to *Lactobacillus*, one isolate to *Pediococcus*, and one isolate to *Neoscardovia*. *Lactobacillus brevis* IBRC‐M 11044, *Pediococcus pentosaceus* IBRC‐M 11045, *Neoscardovia arbecensis* IBRC‐M 4391 4378, and *Lactobacillus pentosus* IBRC‐M 11043 were selected and applied as adjunct culture in producing four treatments of industrial butter. All examined strain treatments showed significant changes in cholesterol level of butter samples. Furthermore in all samples, the cholesterol content was significantly lower than control (*p* < .5). The highest level of cholesterol reduction was achieved in the butter sample prepared by *Lactobacillus pentosus* IBRC‐M 11045. Sensory analysis showed that the butter sample with *Neoscardovia arbecensis* IBRC‐M 4391 4378 was more acceptable than other butter samples. Due to our finding, it is valuable to incorporate these strains in butter production and introduce novel functional butter to market.

## Graphical abstract

Traditional Iranian butter samples were collected from different parts of Iran and were screened for the presence of Lactic acid bacteria. The selected LAB were identified to species level by biphasic approach. The ability of selected LAB to lower the cholestrol both in vitro and in situ was then determined.

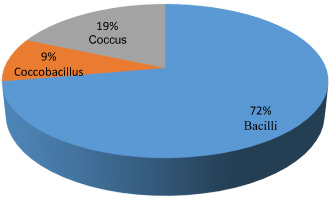

## 1 INTRODUCTION

Traditional butter is produced from yogurt by local farmers in Iran. It is made from churning sour yogurt in goat’s skin. Sheep, goat, or cow’s milk can be used in making yogurt, and during butter production, a little water is added to the yogurt and churned. At the end of churning period, buttermilk is separated, which used to prepare some traditional products locally named “Kashk” and “Doogh.” The remaining butter grains are washed with cold water, while the granules are kneaded by hand. Sometimes, a small amount of salt is added to granules before kneading (KuSak & Avsar, 2010).

Considering the high nutritional value of butter, extensive studies were undertaken to improve qualitative and quantitative properties focusing on cholesterol reduction. One of the reasons for doubling the price of butter from 2015 to 2018 could be a “re‐profile” of butter as a healthy food product (Parmar et al., 2021). Different methods were proposed to reduce cholesterol in foods including extraction, distillation, absorption, enzymatic conversion, or hybrid methods (Mortensen, 2011). None of the mentioned approaches has gained practical success due to high operating costs and low cholesterol volatility (Mortensen, 2011). On the other hand, the approach based on the increased awareness of health benefits is attributed to probiotic products, particularly their ability to reduce serum cholesterol (Ishimwe et al., 2015).

Probiotic foods such as dairy products were classically defined as “foods containing live microorganisms believed to actively enhance health by microbiota balance improvement in gut (Tamime, 2008).” Today, a tremendous increase is shown in microbial species incorporated into probiotic dairy products (e.g., pasteurized milk, ice‐cream, fermented milks, cheeses, and infant milk powder). However, fermented foods remain the primary source of probiotic organisms. Among fermented milk products, yogurt is considered the most crucial mean/medium to deliver probiotic organisms (Tamime, 2008).

Many attempts have been made to identify isolated bacterial strains from traditional dairy products in Iran for using as a starter (Edalatian et al., 2012; Ghiyamati Yazdi, 2012; Milani et al., 2012). However, additional studies on strains’ characterization isolated from traditional dairy products such as stability to acid and bile salts’ conditions can select appropriate probiotic strains to increase health level and reduce cholesterol in butter.

Kim et al., 2021, isolated lactic acid bacteria (LAB) from a traditional fermented Korean food, *kimchi*. LRCC5307 strain was isolated and showed a 74.5% decrease in cholesterol with 0.2% bile salts. After producing butter with LRCC5307 strain as an adjunct culture, it showed 8.74 Log CFU (colony‐forming units)/g viable cells, pH 5.43, and a 11% decrease in cholesterol (Kim et al., 2021). Albano et al. (2018), examined the lowering cholesterol ability in 58 probiotic strains and applied the selected strains in cheese preparation. All strains were able to reduce cholesterol in cheese. *Lactobacillus paracasei* and *Enterococcus lactis* had the highest reduction (23%) (Albano et al., 2018). Ding et al. identified strains by screening LAB from local yogurt. The strains were able to reduce cholesterol more than 60% in a liquid culture medium. *Lactobacillus plantarum* LP3 had the highest cholesterol reduction (73.3%).

This study aimed to investigate the possibility for developing and introducing an adjunct starter with probiotic properties to produce functional butter with low cholesterol and acceptable physicochemical properties.

## 2 MATERIALS AND METHODS

### 2.1 Samples

Ten samples of traditional Iranian butter were obtained from local farms located in Ardebil, Semnan, and Gorgan. All samples were collected according to ISO 707 in 250 ml sterile bottles which were transported to the laboratory under refrigeration (4°C) within 36 h (Anon, 2008).

### 2.2 Chemical analyses

All butter samples were analyzed for moisture, nonfat solids, and fat contents (ISO 8851‐1, ISO 8851‐2, and ISO 8851‐3), acid value (American Oil Chemists’ Society (AOCS) Cd 3d‐63), iodine value (AOCS Cd 1‐25), saponification value (AOCS Cd 3‐25), peroxide value (AOCS Cd 8‐53), and pH (measured by pH meter model Inolab 720, Germany).

### 2.3 Isolation and identification of isolates

Twenty‐five grams of each sample was inoculated in 100 ml MRS broth (deMan, Rogosa, and Sharpe, HiMedia, Mumbai, India) at 37°C, M17 broth at 40°C, and MRS with L‐Cysteine hydrochloride and L‐Mupirocin at 37°C incubated, for 24–48 h in anaerobic and aerobic conditions until growth was observed. Then, decimal dilutions were directly prepared in a 0.1% ringer. Isolation and colony counts were made on the following media: MRS agar, M17 agar, M1712 agar (HiCrome Nickels and Leesment Medium), and MRS + L‐Cysteine hydrochloride + L‐Mupirocin. Finally, plates were anaerobically incubated, and aerobically at 30, 37, and 40°C. The incubation period varied between 24, 48, and 72 h depending on bacterial groups. Counting was conducted only for those plates involving 30–300 colonies and from plates corresponding to the highest dilution. Four to five different colonies (due to shape, size, and color) were randomly selected. The selected colonies were regrown two or three times on the same media. A single colony from each plate was examined by Gram staining, catalase production, and microscopic morphology. Finally, only Gram‐positive, catalase‐negative isolates were considered, and long‐term conservation of purified isolates was carried out in a mixture of MRS broth, M17 broth, and MRS+ L‐Cysteine hydrochloride with sterile glycerol (30%) and stored at −70°C (Sagdic et al., 2002).

### 2.4 Acid tolerance

Acid tolerance of isolates was studied by inoculating each microorganism on appropriate medium. Then, the pH of all media was adjusted to 3.0 with aliquot from each dilution by HCl, which was incubated at an appropriate temperature (30, 37, and 40°C) for 2 h. Serial dilutions were performed and grown on their related medium. The strains capable of growing to >10^7^ CFU (colony‐forming units)/ml after 24 h were considered acid‐resistant strains (Liong & Shah, 2005).

### 2.5 Bile tolerance

The selected isolates were examined for their ability to grow in appropriate medium supplemented with and without bile salts 0.3% (oxgall, Sigma‐Aldrich, St. Louis, MO, USA), the latter was considered as control. The inoculated media were incubated at an appropriate temperature, and the growth was monitored by measuring the absorbance at 620 nm (A620) before and after 8 h of incubation. For quantifying growth inhibition of examined isolates by bile, the inhibition coefficient was calculated, using the following formula:

$$\mathrm{Cin}=\frac{\left(T8-T0\right)\text{control}-\left(T8-T0\right)\text{treatment}}{\left(T8-T0\right)\text{control}}$$

Where T8 denotes control‐ optical density of culture broth without oxgall after 8 h incubation. T0 denotes control‐ optical density of culture broth without oxgall before incubation. T8 denotes treatment‐ optical density of broth involving oxgall after 8 h incubation, to treatment‐ optical density of broth involving oxgall before incubation. Isolates whose inhibitory coefficient is equal to or <0.5 are considered bile‐tolerant isolates (Goderska & Czarnecki, 2007).

### 2.6 Assimilation of cholesterol

Water‐soluble cholesterol was filter‐sterilized and added at a final concentration of 300 μg/ml to a MRS broth tube involving 0.3 g/100 ml oxgall. The tubes were inoculated with each selected strain (at 1 ml/100 ml) and incubated at 37°C for 2, 4, 9, and 24 h (bacterial turbidity was adjusted in phosphate‐buffered saline (PBS) equivalent to 0.5 McFarland). After incubation, the inoculated medium was centrifuged at 10,000 g for 10 min. Measuring absorbance at 546 nm (A546) using a spectrophotometer, the cholesterol‐lowering ability was determined (Pereira & Gibson, 2002). Using the following equation, the ability of selected strains to remove cholesterol from medium was calculated:

$$A=\frac{B-C}{B}\times 100$$

where *A* = % removed cholesterol, *B* = Control absorbance at time 0 min, *C* = Examined strain absorbance.

### 2.7 Identification of selected strains by the molecular method

Molecular techniques identified strains showing the high ability of cholesterol reduction and tolerance to bile and acid. The cell mass of freshly grown selected isolates was harvested, and their DNA was extracted using High yield DNA Purification Kit (CinnaGen Molecular Biology and Diagnostic, Iran).

Polymerase chain reactions (PCRs) were carried out in a thermocycler (Bio‐Rad, USA) in a total volume of 90 μl containing 9 μl buffer, 4.5 μl of each primer 27f (5'‐AGAGTTTGATCM TGGCTCAG‐3') and 1492r (5'‐GGTTACCTTGTTACGACTT‐3') (concentration 0.4 mM), 1.8 MgCl2 (50 mM), 4.5 μl dimethyl sulfoxide (DMSO) (5% mM), 1.3 μl deoxynucleotide triphosphates (dNTPs) (0.4 mM), 3 μl Taq (3.6 U/90 μl), 3 μl template DNA, and the remaining volume of distilled water running under the following temperature program: initial denaturation of DNA for 3 min at 95°C, 25 cycles of 45 s at 95°C, 45 s at 45–59°C, and 1.5 min at 72°C; and final extension for 10 min at 72°C. Three microliter (μl) aliquots of the PCR products with 1 μl buffer were analyzed by electrophoresis using a 0.8% (w/v) agarose gel in Tris acetic acid ethylenediaminetetraacetic acid (EDTA) (TAE. 0.5X) buffer at 120 mA (milliamperes) for 50 min.

Determining the PCR product sequence was done through Korean Bioneer Corporation Company. The sequence received from this company was edited and reread using ChromasPro software which was compared to the sequences stored in EzTaxon genomic database. Isolates with 97% or higher similarity in sequences were identified as the same species.

### 2.8 Preparation of butter sample

**FIGURE 1:**
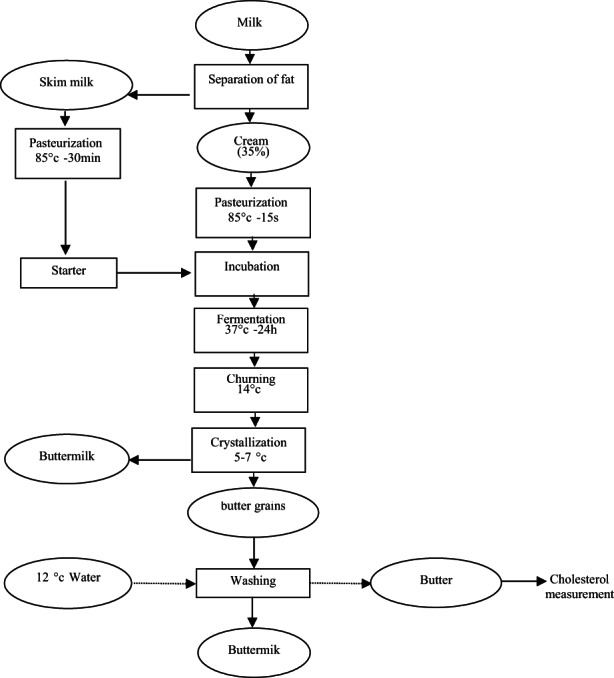
Preparation of butter samples with selected strains

Preparing butter was carried out in Pak dairy factory (Tehran, Iran). The cream was separated from milk by a separator and standardized as 35% cream. It was pasteurized for 15 s at 85°C and divided into five sections. Then, the pasteurized cream was cooled to 37°C and inoculated with selected strains (5% v/v, 10^8^ CFU/ml) at 37°C for 24 h. It was cooled to 14°C and churned. Butter samples (500 g) were packed and kept in refrigerator until the day of the experiment (Figure 1).

### 2.9 Cholesterol measurementand fatty acid compositions

The cholesterol and fatty acid compositions in butter samples (control and treated samples) were determined by gas chromatography (GC), as described by Fletouris et al. (1998). The control sample, which was not inoculated by any isolated strains, was prepared under the same conditions.

### 2.10 Sensory analysis

Some sensory properties of butter samples (appearance, flavor, and consistency) were evaluated by 20 trained panelists (from 23 to 48 years old) using the Hedonic scale test in five levels (1, 2, 3, 4, and 5 by which 1 is very bad and 5 is very good) (Anon, 1997). The mean scores obtained for each sensory property were determined, and using Duncan’s multiple comparison method, the differences among samples and their significance were calculated.

### 2.11 Statistical analysis

To evaluate the effect of added isolated strain on the cholesterol reduction of butter, all experiments were triplicate. The average rating of three values was calculated for each sample (*n* = 3). One‐way analysis of variance (ANOVA) was used to analyze the data following a general linear model in SPSS version 14.0 (SPSS Inc., Chicago, IL, USA). The significance level was set at *p* < .05 to compare among means and obtain the standard deviation (SD) using the Duncan test. All graphs were drawn by Microsoft Excel software (version 2013).

## 3 RESULTS AND DISCUSSION

### 3.1 Chemical analyses

Table 1 shows the physicochemical properties of all treatments. Some samples’ fat content (1, 5, 6, and 7) was lower compared to what was reported in the most conventional butters. The highest fat content (89.5%) was found in sample 8. Mean pH values in samples were similar to those reported in previous studies on butter (Sagdic et al., 2002).

**TABLE 1:** Physicochemical characteristics of traditional butter samples

Sample	Fat (%)	Nonfat solid (%)	Solid (%)	Moisture (%)	pH	Acid value‐ Oleic	Iodine value	Saponification value	Peroxide value
1	80 ± 0.00	0.45 ± 0.11	80.45 ± 0.11	19.55 ± 3.77	3.76 ± 0.01	0.21 ± 0.01	28.42 ± 0.01	225.5 ± 0.5	0.2 ± 0.00
2	85 ± 0.00	2.3 ± 0.10	87.3 ± 0.10	12.7 ± 0.10	4.56 ± 0.02	0.65 ± 0.00	29.19 ± 0.11	223.84 ± 0.56	0.6 ± 0.10
3	85 ± 1.00	3.17 ± 0.02	88.17 ± 1.02	11.83 ± 1.02	4.05 ± 0.00	0.62 ± 0.02	34.26 ± 0.20	219.91 ± 0.09	0.5 ± 0.00
4	85 ± 0.00	2.48 ± 0.13	87.48 ± 0.13	12.52 ± 0.13	3.79 ± 0.15	0.31 ± 0.00	32.50 ± 0.50	218.8 ± 0.00	0.4 ± 0.00
5	81 ± 0.00	1.45 ± 0.45	82.45 ± 0.45	17.55 ± 0.45	3.77 ± 0.08	0.20 ± 0.00	33.5 ± 0.00	216.51 ± 0.50	0.2 ± 0.00
6	81.5±0.50	1.75 ± ± 0.75	83.25 ± 0.25	16.75 ± 0.25	3.97 ± 0.15	0.5 ± 0.00	30.45 ± 0.55	226.64 ± 0.65	0.2 ± 0.00
7	81 ± 1.00	0.15 ± 0.07	81.14 ± 1.05	18.86 ± 1.05	4.79 ± 0.25	0.15 ± 0.00	30.96 ± 0.00	218.8 ± 0.00	0.2 ± 0.00
8	89.5 ± 0.20	1.85 ± 0.30	91.35 ± 0.10	8.65 ± 0.10	4.95 ± 0.05	1.00 ± 0.00	33.75 ± 0.00	225.52 ± 0.00	0.2 ± 0.00
9	84.5 ± 0.50	0.82 ± 0.50	85.32 ± 0.06	14.68 ± 0.06	4.57 ± 0.01	0.34 ± 0.00	36.80 ± 0.00	216.55 ± 0.00	0.2 ± 0.00
10	85.20 ± 0.30	0.60 ± 0.30	85.77 ± 0.02	14.23 ± 0.02	4.84 ± 0.02	0.37 ± 0.03	37.31 ± 0.00	212.06 ± 0.00	0.2 ± 0.00

*Note*: Mean ± standard deviation (SD).

More moisture content in traditional butter is probably due to the difference in the processing equipment and the conditions used to prepare the butter. In traditional methods, butter granules are formed in fermented milk, which has a higher moisture content than the raw materials (cream) used to make industrial butter. Furthermore, due to high dry matter, particularly proteins in granules, the strong binding of proteins with water around butter granules prevents the outflow of water during kneading (Nielsen & Ullum, 1989).

Sagdıc et al. (2004) reported moisture and fat contents of 15.20% and 82.90%, respectively, in traditional butter produced in Turkey, whose production method was similar to traditional Iranian butter.

The chemical properties of butters prepared with selected strains are mentioned in Table 2. Statistically, there was significant difference (*p <* .05) between moisture, fat, nonfat dry matter, pH, acid value‐ oleic, iodine index, and soap index among the samples. According to the existing laboratory conditions for the preparation of butter samples, the moisture and fat contents of the samples did not match the standard of butter preparation. The results of chemical properties obtained from this study are consistent with the results of previous studies (Sagdic et al., 2002).

**TABLE 2:** Chemical characteristics of butters prepared with selected strains

Samples	Fat (%)	Nonfat solid (%)	Solid (%)	Moisture (%)	pH	Acid value‐ Oleic	Iodine value	Saponification value	Peroxide value
Control	69 ± 0.00^a^	1.3 ± 0.06^ab^	70.30 ± 0.05^a^	29.70 ± 0.05^b^	6.55 ± 0.05^d^	0.28 ± 0.00^a^	29.00 ± 0.10^a^	224.8 ± 0.65^a^	–
IBRC‐M 11044 *Lactobacillus brevis*	71 ± 0.00^b^	1.48 ± 0.07^bc^	72.48 ± 0.07^b^	27.51 ± 0.07^a^	4.27 ± 0.02^b^	0.36 ± 0.00^c^	31.72 ± 0.00^d^	225.15 ± 0.65^a^	–
IBRC‐M 11045 *Pediococcus pentosaceus*	71.16 ± 0.28^b^	1.63 ± 0.15^c^	72.8 ± 0.03^bc^	27.20 ± 1.17^a^	4.20 ± 0.02^ab^	0.38 ± 0.01^c^	30.54 ± 0.14^c^	226.64 ± 0.00^b^	‐
IBRC‐ M 4391 *Neoscardovia arbecensis*	69.67 ± 0.57^a^	1.20 ± 0.00^a^	70.87 ± 0.58^a^	29.13 ± 0.58^b^	4.37 ± 0.02^c^	0.33 ± 0.01^b^	29.44 ± 0.00^b^	227.4 ± 0.64^bc^	–
IBRC‐M 11043 *Lactobacillus pentosus*	72.17 ± 0.76^c^	1.43 ± 0.06^b^	73.6 ± 0.72^c^	26.7 ± 0.72^a^	4.15 ± 0.03^a^	0.41 ± 0.01^d^	31.55 ± 0.14^d^	227.76 ± 0.00^c^	–

*Note*: Mean ± standard deviation (SD).

Values in rows with different letters are significantly different (P < .05).

### 3.2 Isolation and identification

**FIGURE 2:**
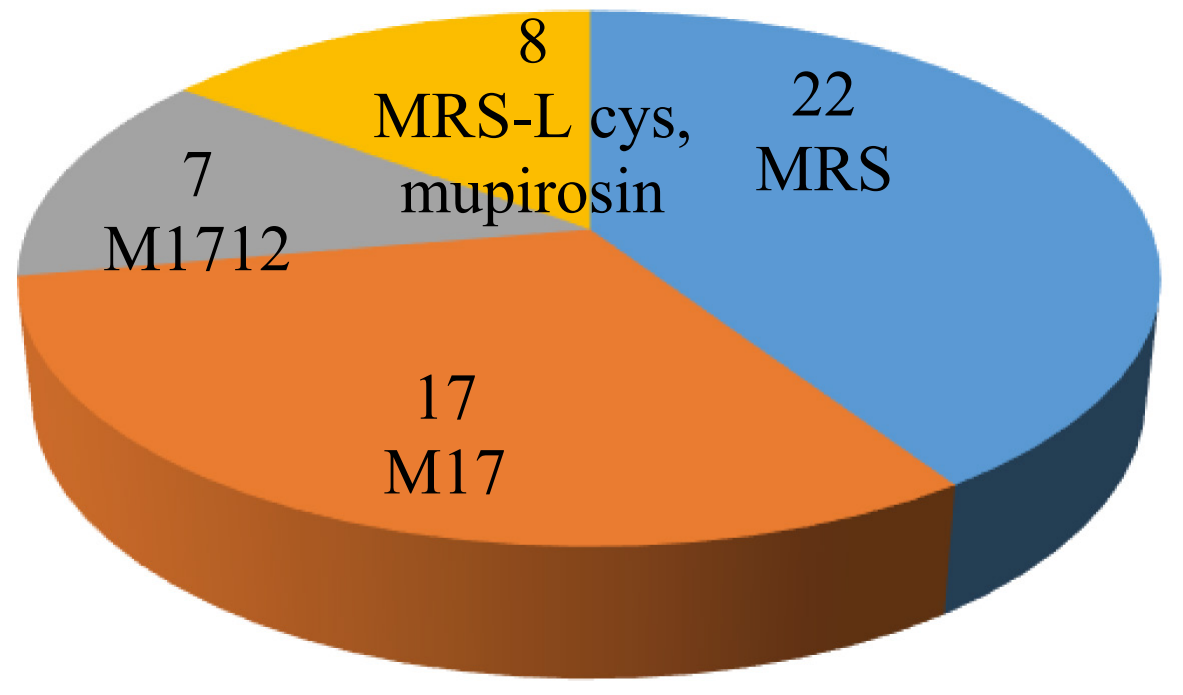
Mean count and frequency of occurrence of bacterial isolates

**FIGURE 3:**
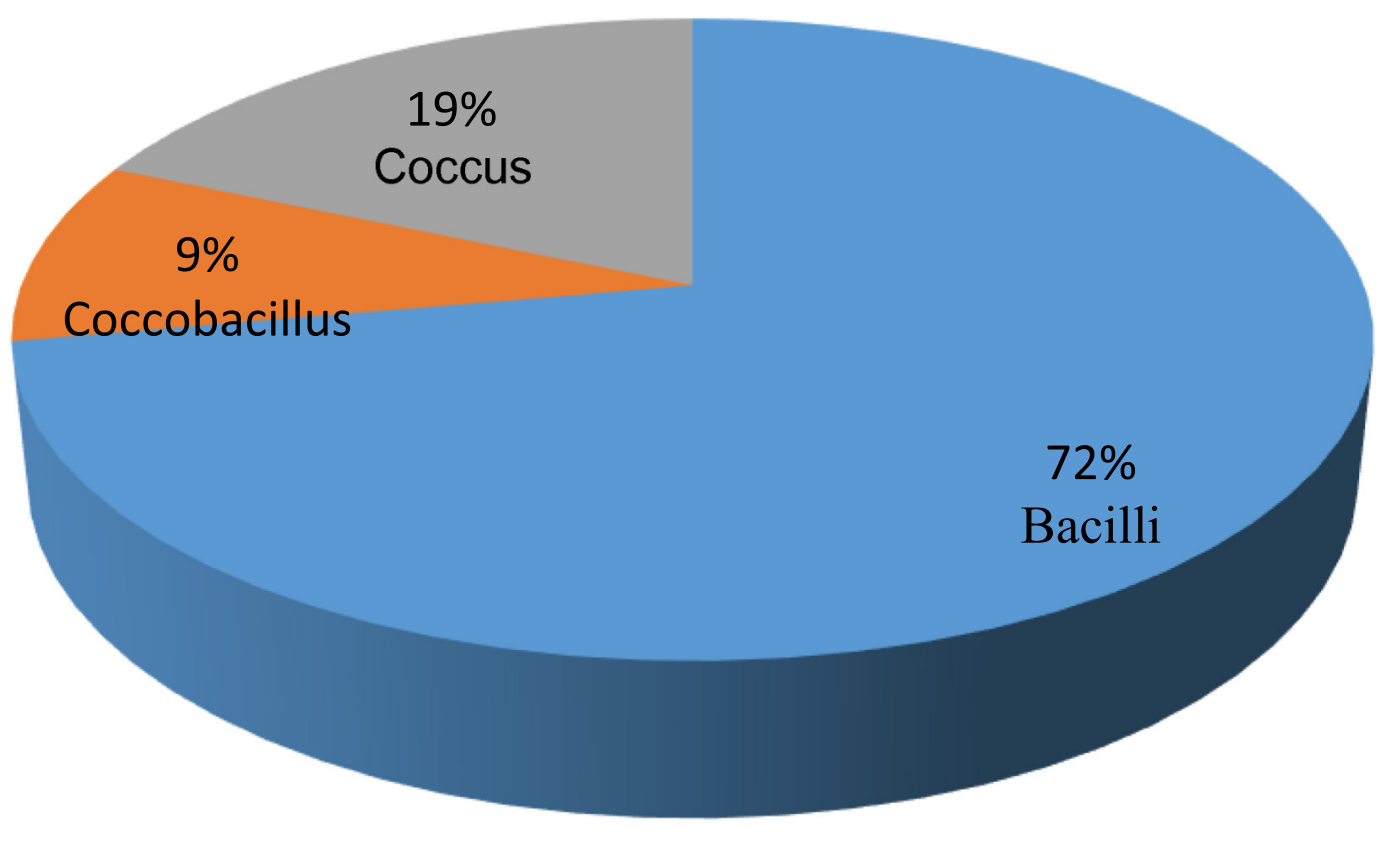
Types of bacteria observed based on appearance

Fifty‐four isolates from 10 samples of traditional butter were identified (Figures 2 and 3). All strains were recorded as catalase‐negative and Gram‐positive cocci in pairs or long chains, bacilli in pairs or chains, and coccobacilli.

### 3.3 Acid tolerance

The effect of acid on isolates is shown in Table 3. All strains showed the tolerance to pH 3.0 for 2 h, despite variations in the degree of viability.

**TABLE 3:** The effect of acid on lactic acid bacteria (LAB) isolates

Isolate no.	Acid‐tolerant (Log cfu/ml)	Isolate no.	Acid‐tolerant (Log cfu/ml)
1	6.52 ± 0.01	28	6.61 ± 0.07
2	6.41 ± 0.03	29	6.75 ± 0.01
3	6.29 ± 0.03	30	5.95 ± 0.12
4	6.25 ± 0.03	31	6.56 ± 0.02
5	6.41 ± 0.01	32	6.44 ± 0.03
6	5.08 ± 0.01	33	6.00 ± 0.07
7	6.48 ± 0.03	34	6.20 ± 0.03
8	6.33 ± 0.01	35	6.56 ± 0.05
9	6.21 ± 0.01	36	6.34 ± 0.05
10	7.26 ± 0.04	37	6.51 ± 0.02
11	6.16 ± 0.06	38	6.25 ± 0.02
12	6.22 ± 0.03	39	6.31 ± 0.03
13	7.09 ± 0.05	40	6.93 ± 0.06
14	6.03 ± 0.01	41	6.45 ± 0.02
15	6.47 ± 0.01	42	5.91 ± 0.09
16	6.69 ± 0.01	43	6.45 ± 0.03
17	6.35 ± 0.03	44	7.46 ± 0.09
18	6.31 ± 0.04	45	6.03 ± 0.58
19	6.26 ± 0.01	46	6.10 ± 0.07
20	6.50 ± 0.03	47	6.37 ± 0.03
21	6.49 ± 0.01	48	6.24 ± 0.03
22	5.89 ± 0.12	49	7.42 ± 0.03
23	6.93 ± 0.05	50	6.57 ± 0.01
24	6.78 ± 0.1	51	6.65 ± 0.06
25	6.05 ± 0.02	52	6.44 ± 0.06
26	6.17 ± 0.1	53	6.25 ± 0.04
27	6.30 ± 0.07	54	6.50 ± 0.07

*Note*: Mean ± standard deviation (SD).

Isolates with code nos. 4331, 4335, 4368, and 4373 were the most acid‐tolerant strains, with more than 7 (log CFU/ml) after incubation for 2 h at pH 2.0, while isolates with code nos. 4326, 4344, 4352, and 4366 were the most acid‐sensitive strains.

The ability to survive and grow at low pH environment is a key characteristic of probiotic bacteria. Acidic stress may inhibit bacterial growth by acidifying the cytoplasm, increasing energy consumption required for maintenance of intracellular pH, and inhibiting enzymatic reactions (Shabala et al., 2006). In many acid‐tolerant fermentative bacteria, the intracellular pH decreases as the extracellular pH decreases during growth in order to maintain a constant pH gradient rather than a constant intracellular pH. Generating a large proton gradient is disadvantageous for fermentative LAB, because proton translocation consumes energy, and anaerobic organisms gain significantly less energy from sugar metabolism than what aerobes gain. Furthermore, a large proton gradient results in the accumulation of organic acid anions in the cytosol. There are several possible mechanisms by which a bacterium can regulate the intracellular pH, but the most important mechanism in fermentative bacteria appears to be the proton‐translocating p‐type adenosine triphosphatase (ATPase) (Siegumfeldt et al., 2000). Several proteins that are able to protect or repair macromolecules such as DNA are also effective in acid tolerance. It is believed that acidification in the cell reduces purine and pyrimidine in DNA (Almedia et al., 2015).

The bacterial cells are affected by pH during their enzymes’' function and nutrients’ transfer into the cell. When bacteria are present in acidic environments, they should prevent hydrogen ions from entering into the interior of the cell, or simultaneously release the equivalent hydrogen ions from the cytoplasm because neutral conditions are needed to maintain vital components such as DNA and adenosine triphosphate (ATP) in a cell. Otherwise, bacterium consumes energy which quickly drains cell’s energy and weakens it, eventually leading to cell death (Jay et al., 2006).

Also, the difference of macromolecule components of membrane and cell wall in *Lactobacillus* can maintain the cells during different stresses. For instance, a low pH can cause a change in cell wall fatty acids of *Lactobacillus casei* (Fozo et al., 2004).

### 3.4 Bile tolerance

Tolerance to bile allows LAB to survive in small intestine. By analyzing coefficients of growth inhibition (Table 4), it can be concluded that isolated strains coded 4328, 4334, 4336, 4348, 4358, 4363, 4367, 4373, 4377, and 4378 should be considered as bile‐tolerant because their growth inhibition coefficient is <0.5 (Goderska & Czarnecki, 2007).

**TABLE 4:** Coefficient of inhibition in medium with oxgall

	Optical density in T0		Optical density in T8		Coefficient of inhibition
	Control	The broth containing oxgall	Control	The broth containing oxgall	
1	0.036 ± 0.005	0.10 ± 0.00	0.19 ± 0.01	0.075 ± 0.005	1.163 ± 0.03
2	0.098 ± 0.04	0.042 ± 0.02	0.836 ± 0.05	0.045 ± 0.03	1.00 ± 0.06
3	0.016 ± 0.005	0.027 ± 0.01	0.133 ± 0.04	0.046 ± 0.005	0.836 ± 0.03
4	0.096 ± 0.04	0.062 ± 0.006	0.433 ± 0.07	0.053 ± 0.03	1.009 ± 0.08
5	0.083 ± 0.01	0.07 ± 0.01	0.656 ± 0.05	0.095 ± 0.04	0.958 ± 0.05
6	0.016 ± 0.005	0.034 ± 0.01	0.15 ± 0.01	0.048 ± 0.02	0.894 ± 0.05
7	0.473 ± 0.06	0.466 ± 0.07	2.426 ± 0.02	0.916 ± 0.07	0.769 ± 0.02
8	0.16 ± 0.07	0.09 ± 0.01	0.866 ± 0.05	0.5 ± 0.00	0.417 ± 0.04
9	0.136 ± 0.07	0.153 ± 0.09	1.133 ± 0.15	0.216 ± 0.05	0.931 ± 0.08
10	0.033 ± 0.005	0.046 ± 0.005	0.333 ± 0.03	0.136 ± 0.005	0.699 ± 0.01
11	0.05 ± 0.01	0.07 ± 0.01	0.103 ± 0.01	0.088 ± 0.008	0.648 ± 0.04
12	0.04 ± 0.02	0.036 ± 0.02	0.446 ± 0.06	0.25 ± 0.07	0.479 ± 0.07
13	0.153 ± 0.005	0.103 ± 0.01	0.446 ± 0.04	0.1 ± 0.01	1.012 ± 0.03
14	0.073 ± 0.005	0.067 ± 0.01	0.091 ± 0.007	0.076 ± 0.01	0.511 ± 0.01
15	0.17 ± 0.06	0.253 ± 0.05	1.796 ± 0.72	0.586 ± 0.05	0.764 ± 0.09
16	0.043 ± 0.005	0.096 ± 0.005	0.23 ± 0.02	0.15 ± 0.01	0.715 ± 0.07
17	0.12 ± 0.01	0.11 ± 0.01	0.59 ± 0.06	0.036 ± 0.005	1.162 ± 0.008
18	0.02 ± 0.00	0.01 ± 0.00	0.569 ± 0.03	0.042 ± 0.004	0.940 ± 0.008
19	0.103 ± 0.07	0.07 ± 0.02	0.476 ± 0.06	0.18 ± 0.06	0.723 ± 0.04
20	0.16 ± 0.02	0.08 ± 0.03	0.483 ± 0.06	0.186 ± 0.01	0.664 ± 0.02
21	0.146 ± 0.05	0.26 ± 0.08	2.16 ± 0.55	0.473 ± 0.03	0.884 ± 0.05
22	0.023 ± 0.005	0.026 ± 0.005	0.61 ± 0.03	0.12 ± 0.01	0.840 ± 0.02
23	0.076 ± 0.04	0.055 ± 0.03	0.356 ± 0.05	0.066 ± 0.03	0.957 ± 0.02
24	0.05 ± 0.00	0.083 ± 0.005	0.216 ± 0.02	0.03 ± 0.00	1.327 ± 0.07
25	0.153 ± 0.05	0.33 ± 0.026	1.683 ± 0.10	0.546 ± 0.04	0.859 ± 0.03
26	0.013 ± 0.005	0.016 ± 0.005	0.496 ± 0.04	0.306 ± 0.03	0.400 ± 0.01
27	0.053 ± 0.005	0.08 ± 0.01	0.313 ± 0.03	0.056 ± 0.01	1.091 ± 0.06
28	0.053 ± 0.005	0.096 ± 0.005	0.246 ± 0.05	0.063 ± 0.01	1.183 ± 0.07
29	0.1 ± 0.00	0.093 ± 0.01	0.36 ± 0.05	0.05 ± 0.02	1.191 ± 0.18
30	0.116 ± 0.01	0.123 ± 0.04	0.243 ± 0.01	0.07 ± 0.02	1.427 ± 0.2
31	0.126 ± 0.02	0.11 ± 0.01	0.273 ± 0.04	0.123 ± 0.005	0.904 ± 0.03
32	0.033 ± 0.005	0.086 ± 0.005	0.293 ± 0.03	0.123 ± 0.02	0.860 ± 0.07
33	0.046 ± 0.005	0.033 ± 0.005	0.486 ± 0.01	0.009 ± 0.001	1.055 ± 0.01
34	0.1 ± 0.00	0.083 ± 0.005	0.506 ± 0.03	0.086 ± 0.005	0.990 ± 0.02
35	0.13 ± 0.01	0.12 ± 0.03	0.72 ± 0.07	0.443 ± 0.07	0.454 ± 0.04
36	0.054 ± 0.005	0.063 ± 0.005	0.106 ± 0.01	0.073 ± 0.005	0.799 ± 0.04
37	0.033 ± 0.005	0.063 ± 0.01	0.156 ± 0.01	0.069 ± 0.01	0.95 ± 0.04
38	0.053 ± 0.005	0.073 ± 0.01	0.39 ± 0.05	0.08 ± 0.01	0.980 ± 0.01
39	0.103 ± 0.06	0.11 ± 0.06	1.68 ± 0.27	0.933 ± 0.07	0.475 ± 0.01
40	0.0133 ± 0.005	0.11 ± 0.03	1.29 ± 0.06	0.16 ± 0.01	0.961 ± 0.01
41	0.66 ± 0.00	0.66 ± 0.00	1.44 ± 0.00	0.62 ± 0.05	1.051 ± 0.06
42	0.426 ± 0.005	0.396 ± 0.01	0.687 ± 0.01	0.313 ± 0.05	1.329 ± 0.2
43	0.366 ± 0.07	0.406 ± 0.05	0.953 ± 0.05	0.736 ± 0.05	0.438 ± 0.03
44	0.156 ± 0.07	0.206 ± 0.05	0.803 ± 0.05	0.383 ± 0.02	0.726 ± 0.03
45	0.096 ± 0.05	0.12 ± 0.06	0.776 ± 0.02	0.19 ± 0.04	0.897 ± 0.01
46	0.046 ± 0.005	0.043 ± 0.005	0.873 ± 0.04	0.14 ± 0.03	0.881 ± 0.04
47	0.106 ± 0.02	0.106 ± 0.01	1.43 ± 0.06	0.353 ± 0.04	0.813 ± 0.02
48	0.03 ± 0.00	0.027 ± 0.003	0.85 ± 0.05	0.084 ± 0.02	0.930 ± 0.02
49	0.106 ± 0.06	0.11 ± 0.07	0.833 ± 0.08	0.476 ± 0.08	0.494 ± 0.02
50	0.03 ± 0.00	0.026 ± 0.01	2.4 ± 0.24	0.725 ± 0.07	0.705 ± 0.004
51	0.356 ± 0.05	0.33 ± 0.05	1.69 ± 0.75	0.486 ± 0.08	0.858 ± 0.08
52	0.033 ± 0.005	0.043 ± 0.005	0.926 ± 0.05	0.143 ± 0.005	0.887 ± 0.007
53	0.031 ± 0.01	0.029 ± 0.001	0.4 ± 0.02	0.034 ± 0.01	0.569 ± 0.08
54	0.033 ± 0.005	0.03 ± 0.005	0.077 ± 0.03	0.059 ± 0.02	0.529 ± 0.07

*Note*: Mean ± standard deviation (SD).

Bile is a green‐yellow alkaline liquid, secreted by the liver cells, which is involved in fat digestion. Bile is a solution of bile acids (weak organic acids) and their salts (containing amino acid, taurine and glycine), cholesterol, phospholipids, and bile pigments. Bile causes folding and/or denaturation of cell wall proteins, DNA and RNA degradation, pH reduction, osmotic stress, and oxidation, as well as bacterial cell wall damage. As a result, the ability to survive, grow, and reproduce in such a situation is used as a benchmark when assessing probiotic strains’ potential (Lv et al., 2017).

Despite the proteomics studies and the expression of several genes reported for the resistance of probiotic strains to bile, the mechanism of tolerating probiotics to bile salts is still not completely clear. The active release of acids or bile salts to the outside, bile salts’ hydrolysis, and changes in the composition of the bacterium cell membrane and the cell wall are believed to be the most common mechanisms for bile resistance in both *Lactobacillus* and *Bifidobacterium* (Kumar et al., 2006; Lv et al., 2017; Pfeiler & Klaenhammer, 2009). Bacterial bile salt hydrolase (BSH), which controls bile salts’ deconjugation reaction, is believed to take an active part in abating the bile salts’ toxicity. The amino acid released by deconjugation can be further utilized as carbon and nitrogen sources to benefit bacterial sustenance and survival. BSH is an intracellular, nonallosteric enzyme which is nonsensitive to oxygen; the optimum pH is 5–6, and the activity of BSH is associated with biomass density (Niamah et al., 2017).

Besides, bacterial cells’ colony shape and morphology play an essential role in the resistance to biliary salts in probiotics. Suskovic et al. (2000) reported that the *Lactobacillus acidophilus* M92 colonies are smooth and rough, and the rough type is more sensitive to biliary salts. They suggested that this difference was not due to a genetic mutation in the bacteria due to environment’s phototypic response. Smooth colonies have a compact structure with short chains, while rough colonies are longer and more vulnerable to biliary salts. Increasing the production of exopolysaccharides in *Bifidobacterium* affected by biliary salts has a protective role for the cell wall (Mozzi et al., 2016). Also, in proteomics studies by Burns et al. (2010), they showed resistance to bile salts in *Lactobacillus delbrueckii*, which is directly related to the ability to code enzymes involved in the production of exopolysaccharides.

### 3.5 Assimilation of cholesterol

Table 5 shows cholesterol reduction percentages in media for all strains after 2, 4, 6, and 24 h incubation. All examined strains were able to assimilate cholesterol from 10% to 75% in the media. As depicted in Table 5, strain 4367 assimilated the highest cholesterol level during 2 and 4 h, while strain 4348 exhibited the highest cholesterol reduction activity after 6 and 24 h.

**TABLE 5:** Cholesterol reduction in media for all the strains after 2, 4, 6, and 24 h incubation

Isolates	Time (h)	Cholesterol reduction (%)
4328	2	33.33
	4	36.18
	6	43.62
	24	59.00
4334	2	12.70
	4	27.45
	6	33.33
	24	38.52
4336	2	21.82
	4	22.62
	6	26.92
	24	29.16
4348	2	36.83
	4	43.18
	6	65.62
	24	75.00
4358	2	18.48
	4	33.33
	6	34.77
	24	39.33
4363	2	29.16
	4	38.54
	6	55.78
	24	59.55
4367	2	37.83
	4	45.15
	6	55.12
	24	60.45
4373	2	12.70
	4	27.45
	6	30.5
	24	47.63
4377	2	21.14
	4	29.57
	6	39.84
	24	47.15
4378	2	33.65
	4	44.44
	6	58.33
	24	65.60

Kaur et al. (2002) reported that *Lactobacillus reuteri* reduced triglyceride by 40% and cholesterol by 38%. Feeding mice with this strain for 7 days increased the high‐density lipoprotein/lipoprotein (HDL/LDL) ratio by 20%.

Liong and Shah (2005) suggested that the *Lactobacillus* cell wall absorbed the environmental cholesterol by studying the fatty acid profile in *Lactobacilli*, especially palmitic acid, stearic acid, total saturated and unsaturated fatty acids with and without cholesterol.

### 3.6 Molecular identification of selected strains

The PCR was performed to amplify 16S rRNA (ribosomal RNA) region after DNA extraction of selected strains. After transferring the PCR product onto the gel, they had a length from 1400 bp to 1500 bp. Due to sharp bands’ formation in the electrophoresis gel, the extracted DNA’s quantity and quality were confirmed and amplicons were sent to *Bioneer Corporation* for sequencing. The sequence received from this company was edited and reread using ChromasPro software and compared with the sequences recorded in the EzTaxon genomic database, and its similarity to different registered strains was studied (Table 6).

**TABLE 6:** Molecular identification of selected strains

No	Isolates	Top‐hit taxon	Similarity (%)
1	4328	*Enterococcus durans CECT411*	99.78
2	4334	*Lactobacillus brevis ATCC 14869*	99.79
3	4336	*Pediococcus pentosaceus DSM 20336*	99.79
4	4348	*Enterococcus durans CECT411*	99.85
5	4358	*Lactobacillus brevis ATCC 14869*	99.93
6	4363	*Enterococcus durans CECT411*	99.93
7	4367	*Enterococcus durans CECT411*	99.7
8	4373	*Lactobacillus delbrueckii* subsp*. lactis DSM 20072*	99.77
9	4377	*Lactobacillus pentosus JCM 1558*	99.85
10	4378	*Neoscardovia arbecensis PG 10*	96.76

The Basic Local Alignment Search Tool (BLAST) program on National Center for Biotechnology Information (NCBI) (http://www.ncbi.nlm.nih.gov) initially determined the species with more than 99% similarity compared to 16s rRNA sequences of type strains, and only one isolate had 96.76% similarity. The results indicated that four isolates belonged to *Enterococcus durans*, four isolates to *Lactobacillus*, one isolate to *Pediococcus*, and one isolate to *Neoscardovia* (Table 6).

The similarity among isolated cocci strains was examined as shown in Table 7 using BLAST program (https://blast.ncbi.nlm.nih.gov/Blast.cgi).

**TABLE 7:** Similarity of isolated cocci strains

Isolates	Closed match	Query cover (%)
4348	*Enterococcus durans CECT411*	100
4363	*Enterococcus durans CECT411*	98
4367	*Enterococcus durans CECT411*	96
4328	*Enterococcus durans CECT411*	99

The isolates with 4363 and 4328 codes had 98% and 99% similarity with 4348 isolates, respectively, and the isolate coded 4367 had 96% similarity with isolate 4348. In other words, three strains are in different subtypes.

*Enterococcus*’s presence in fermented products has led to aroma development is still debatable. Some researchers believe that large amounts of *Enterococcus* produce disintegration and undesirable effects in some dairy products (Erdogrul & Erbilir, 2006). On the other hand, many reports point to this strain’s desired effect on the aroma production and quality of dairy products (Graham et al., 2019).

Considering Table 6, among ten selected strains capable of lowering cholesterol and resistance to acidic environments and bile salts, four strains belong to genus *Lactobacillus*, four strains belonging to *Enterococcus*, one strain belonging to *Pedicococcus*, and one strain belonging to *Neoscardovia*. Ding et al. (2017) identified 115 strains by screening LAB from local yogurt, which belong to four genera *Lactobacillus*, *Enterococcus*, *Lactococcus*, and *Leuconostoc*. *Lactobacillus plantarum*, *Lactobacillus delbrueckii subsp. Lactis*, *Enterococcus durans*, *Enterococcus faecalis*, *and Lactobacillus paracasei*, *Lactococcus lactis* subsp*. lactis* could reduce cholesterol more than 60% in a liquid culture medium. *Lactobacillus plantarum* LP3 had the highest cholesterol reduction (73.3%) (Ding et al., 2017). Iranmanesh et al. (2014) isolated LAB from Ewe’s milk, local yogurt, and buttermilk from different East Azerbaijan regions. Four strains, including *Lactobacillus brevis*, *Lactobacillus pentosus*, *Pediococcus acidilactici*, and *Lactobacillus paracasei*, were able to inhibit the growth of pathogenic bacteria such as *Listeria monocytogenes*, *Salmonella enteritidis*, and *Staphylococcus aureus* and grow in acidic environments as well as bile salts. *Lactobacillus brevis* had the highest cholesterol‐lowering level. Molecular identification results of examined ten strains were similar to the results of Ding et al. (2017) and Iranmanesh et al. (2014).

Lactic acid bacteria (LAB) strains previously reported possessing cholesterol reduction ability belonging to *Enterococcus durans* (Ding et al., 2017), *Lactobacillus brevis* (Iranmanesh et al., 2014), *Pediococcus pentosaceus* (Vidhyasagar & Jeevaratnam, 2013), *Lactobacillus delbrueckii* subsp*. lactis* (Shehata et al., 2016), and *Lactobacillus pentosus* (Iranmanesh et al., 2014).

### 3.7 The analysis results of fatty acid and cholesterol levels in the samples

Due to the results of probiotic properties’ tests (resistance to acid, bile salts, and ability to reduce cholesterol), *Lactobacillus brevis* IBRC‐M 11044, *Pediococcus pentosaceus* IBRC‐M 11045, *Neoscardovia arbecensis* IBRC‐M 4391 4378, and *Lactobacillus pentosus* IBRC‐M 11043 were used to produce industrial butter. Table 8 indicates the analysis of fatty acid and cholesterol levels in samples. The total saturated fatty acids in all samples were less than the control, while the total unsaturated fatty acids were higher than the control. In other words, using selected strains as an adjunct starter reduced the amount of saturated fatty acids in butter and increased the amount of unsaturated fatty acids. The lowest amount of saturated fatty acids and the highest amount of unsaturated fatty acids were observed in the butter sample prepared with *Lactobacillus pentosus* IBRC‐M 11043 compared to control. Total saturated fatty acids in the control and butter sample prepared by *Lactobacillus pentosus* IBRC‐M 11043 were 76.1% and 66.66%, respectively, and the amounts of unsaturated fatty acids were 22.3% and 32.92%, respectively. The results were similar to the results of Bezerra et al. (2017), who investigated the effect of probiotic on the fatty acid profile of cheese, as well as Florence et al. (2012), who studied the effect of probiotic on the fatty acid profile of the local milk and sterilized milk. The percentage of short fatty acids (butyric acid, caproic acid, caprylic acid, and capric acid) in control sample and butter samples prepared with *Lactobacillus brevis*, *Pediococcus pentosaceus*, *Neoscardovia arbecensis*, and *Lactobacillus pentosus* were 12.3%, 4.9%, 8.7%, 15.2%, and 6.6%, respectively. The results of published research on the impact of fermentation on changing fatty acid profile are inconsistent. Santos Junior et al. (2012) have noted that adding starter to dairy products releases fatty acids due to lipolysis, and due to the function of lipases derived from the starter on the positions 1 and 3 of triglycerides and on the other hand, considering the fact that the majority of short‐ and intermediate‐chain fatty acids are at the position 3 of triglycerides in milk fat, release and reduction of short‐chain fatty acids are further increased (Santos Junior et al., 2012). Short‐chain fatty acids have low molecular weight and high solubility, so they can easily enter buttermilk and thus be lower in butter. On the other hand, as the butyric acid and caproic acid are soluble in water, the solubility increase with decreasing pH and the tendency to transfer to buttermilk increases (Fox & McSweeney, 2006). While Ekinci et al. (2008) investigated the effect of cream fermentation with different probiotic bacteria as well as the addition of sunflower, soybean, and hazelnut oils on fatty acids of cream and they concluded that short‐chain fatty acids such as butyric, caproic, and capric acids were significantly affected by culture media incorporation in sour cream and they increased compared to the control group where no fermentation took place. They also reported that the rate of change varied depending on the type of culture used and it was higher for sour cream with *L. acidophilus* than the others. However, unsaturated long‐chain fatty acids were significantly affected by the type of oil added to them (Ekinci et al., 2008).

**TABLE 8:** The analysis results of fatty acid and cholesterol levels in the samples

Fatty acids (FA)	Control	Butter sample prepared with *Lactobacillus brevis* IBRC‐M 11044	Butter sample prepared with *Pediococcus pentosaceus* IBRC‐M 11045	Butter sample prepared with *Neoscardovia arbecensis* IBRC‐M 4391 4378	Butter sample prepared with *Lactobacillus pentosus* IBRC‐M 11043
C4:0	1.6	1.0	2.5	1.8	1.7
C6:0	3.3	1.1	2.0	3.7	1.7
C8:0	1.9	0.7	1.4	2.8	1.0
C10:0	5.5	2.1	2.8	6.9	2.9
C11:0	–	–	0.5	0.2	–
C12:0	6.0	2.6	3.6	6.9	3.5
C13:0	0.3	0.1	0.1	0.2	–
C14:0	16.7	9.8	11.7	16.4	11.4
C15:0	2.2	1.1	1.2	0.8	1.2
C16:0	33.1	37.2	32.0	28.0	32.5
C17:0	0.9	0.4	0.7	0.8	1
C18:0	4.3	10.1	8.3	6.0	9.3
C20:0	–	0.3	0.3	0.3	0.3
C22:0	0.3	0.3	0.1	0.1	0.1
Saturated	76.1	66.8	67.2	74.9	66.6
C10:1c	0.7	0.1	0.4	0.6	0.5
C12:1t	0.2	–	–	–	–
C12:1c	0.2	0.1	0.1	0.3	0.2
C14:1t	–	–	–	0.1	–
C14:1c	1.5	1.5	1.9	1.7	1.7
C15:1	0.3	0.1	0.1	1.4	0.2
C15:2	–	0.2	0.2	0.4	–
C16:1t	0.3	–	–	0.5	–
C16:1c	2.0	0.7	2.1	1.6	1.7
C17:1	0.5	–	0.7	0.5	0.7
C17:2	0.9	–	0.4	0.1	0.2
C18:1t	–	–	–	–	–
C18:1c	12.1	25.8	21.7	14.6	23.9
C18:2t	0.6	–	0.2	0.1	–
C18:2c	2	3.4	2.8	1.8	3
C20:1	0.3	0.6	0.1	0.2	–
C18:3t	–	–	0.5	–	–
C18:3c	–	0.6	0.5	0.4	0.8
C18:3y	–	–	–	–	–
C18:2,9c,11t	–	–	0.1	0.2	–
C18:2,10t,12c	–	–	–	0.1	–
C22:1	0.7	0.1	0.1	–	–
Unsaturated	22.3	33.2	31.9	24.6	32.9
Cholesterol (mg per 100 g)	264.3 ± 0.78^e^	232.2 ± 0.70^c^	206.5 ± 0.20^b^	251.5 ± 0.20^d^	168.6 ± 0.36^a^

Values in rows with different letters are significantly different (P < .05).

Loric acid, myristic acid, and palmitic acid are known as atherogenic factors, and their presence in high level in food has a high risk of cardiovascular diseases (Kim et al., 2006). Total amounts of these three fatty acids for control sample, butter sample prepared with *Lactobacillus brevis*, *Pediococcus pentosaceus*, *Neoscardovia arbecensis*, and *Lactobacillus pentosus* were 55.8, 49.6, 47.3, 51.3, and 47.4, respectively. In other words, fermentation of butter with the examined strains has reduced these fatty acids.

As shown in Table 8, the strains used have led to significant changes in samples’ cholesterol content, and in all samples, cholesterol content was lower than the control. The highest level of cholesterol reduction was achieved in the butter sample prepared by *Lactobacillus pentosus* IBRC‐M 11043. Our finding is consistent to the results expressed by other researchers, which pointed out high ability of lactobacilli in lowering cholesterol.

Albano et al. (2018) examined the ability of 58 probiotic strains to lower cholesterol. They found that *Lactobacillus casei*, *Lactobacillus paracasei*, *Lactobacillus plantarum*, *Enterococcus faecalis*, and *Enterococcus lactis* had the ability to lower cholesterol from 42% to 55% in the liquid culture medium. All strains could reduce cholesterol in cheese; however, *Lactobacillus paracasei* and *Enterococcus lactis* had the highest reduction (23%). There was also no negative effect on the sensory properties of cheese. It was recommended to use these strains to produce functional fermented dairy products (Albano et al., 2018).

The effect of low pH on the increase of cholesterol uptake by LAB has been discussed in studies performed by Rasic et al., 1992; Pereira & Gibson, (2002). Reported that *Lactobacillus fermentum* strain KC5b, isolated from the human gut, was regarded as a candidate probiotic. It maintained viability for 2 h at pH 2 and grew in a medium with 4000 mg of bile acids per liter. This strain was also able to remove a maximum of 14.8 mg of cholesterol per g (dry weight) of cells from the culture medium. Also, Aloğlu and Öner (2006) confirmed that the cholesterol uptake by *Lactobacillus caesium* at pH 4.6 and 3.96 was 4.05% and 25.35%, respectively.

### 3.8 Sensory evaluation

As is evident in Table 9, comparing the average points about appearance, flavor, and consistency shows a significant difference among treatment samples and control (*p* < .05). Butter sample prepared with *Neoscardovia arbecensis* IBRC‐M 4391 4378 gained higher scores for appearance, flavor, and consistency. Oxidation is the main cause of a reduction in the quality and shelf life of high‐fat dairy products. Peroxide is generated by the reaction of oxygen with unsaturated fatty acids, leading to the decomposition of fatty acids and carbonyl production, with unpleasant flavor and aroma in butter (Senel et al., 2011). In this study, undesirable flavor and aroma, such as bitterness and, rancidity, have not been detected by panelists.

**TABLE 9:** Sensory evaluation of butters

Sample	Appearance	Flavor	Consistency
Control	3.34 ± 1.03^a^	2.43 ± 0.87^a^	3.40 ± 0.97^a^
Butter sample prepared with *Lactobacillus brevis* IBRC‐M 11044	3.37 ± 0.9^a^	3.25 ± 0.91^b^	3.28 ± 0.95^a^
Butter sample prepared with *Pediococcus pentosaceus* IBRC‐M 11045	3.65 ± 0.9^ab^	3.62 ± 1.09^b^	3.65 ± 0.78^ab^
Butter sample prepared with *Neoscardovia arbecensis* IBRC‐M 43914378	4.12 ± 1.03^b^	3.65 ± 1.15^b^	4.06 ± 0.94^b^
Butter sample prepared with *Lactobacillus pentosus* IBRC‐M 11043	3.65 ± 1.15^ab^	3.46 ± 1.10^b^	3.68 ± 0.93^b^

Values in rows with different letters are significantly different (P < .05).

### 3.9 Conclusion

In this study, LAB with probiotic and cholesterol‐lowering properties were isolated from traditional Iranian butters and identified to the species level. Due to our findings, it can be concluded that four strains mentioned above are considered suitable candidates as probiotic strains with a high ability to reduce cholesterol in butter. However, *Pediococcus pentosaceus* IBRC‐M 11045 and *Lactobacillus pentosus* IBRC‐M 11043 have a higher potential for cholesterol reduction among these strains. By examining the safety of these highly cholesterol‐lowering strains (production of toxic metabolites, antibiotic resistance) and their technological characteristics, we can recommend these strains to be used commercially in functional butter production.

## CONFLICT OF INTEREST

The authors declare that they have no conflict of interests.

## Data Availability

Research data are not shared.
